# Upper gastrointestinal bleeding: predictors for admission to emergency room

**DOI:** 10.1186/2197-425X-3-S1-A336

**Published:** 2015-10-01

**Authors:** N Melo, C Carvalho, I Pedroto, I Aragão, R Antunes

**Affiliations:** Instituto de Ciências Biomédicas Abel Salazar, University of Porto, Porto, Portugal; Hospital de Santo António, Centro Hospitalar do Porto, Intensive Care Unit, Porto, Portugal; Gastroenterology Department, Hospital de Santo António, Centro Hospitalar do Porto, Porto, Portugal

## Introduction

Upper gastrointestinal bleeding (UGIB) is a frequent cause of emergency department admission. It can be caused by a wide spectrum of pathologies, some of which carry clinically significant morbidity and mortality. To stratify the risk and plan the level of care at admission at Emergency Department is challenging. Several risk factors for adverse outcomes are known and different risk scores are developed and applied to stratify these patients. It is unclear if any of these tools are sufficiently predictive to guide emergency physicians' decisions

## Objectives

To evaluate independent clinical and laboratory variables associated with need for organ support and consequently admission to the Emergency Room. To study the adequacy of AIMS65 and Glasgow-Blatchford Bleeding Score (GBS) in an emergency setting.

## Methods

A retrospective cohort study was conducted in all patients with endoscopically confirmed UGIB admitted in an Emergency Room at tertiary, university hospital between January 2010 and September 2014. Need for organ dysfunction support was defined as need for endotracheal intubation, blood, platelets and coagulation factors transfusion, mechanical ventilation and vasopressor support. Association of independent predictors with need for organ dysfunction support was studied through logistic regression models. Accuracy of risk scores AIMS65 and GBS to predict need of organ dysfunction support was assessed by the area under the curve (AUC).

## Results

A total of 504 patients presented with UGIB to the Emergency Room during the study period; of those 201, (159 men; median age 60 ± 3 years) were included in the study. A total of 44 patients died for an overall mortality rate of 21,9 % and 155 patients (77,1%) needed organ dysfunction support. On multivariate analysis, ischemic cardiopathy, chronic kidney disease, raised urea and low hemoglobin were found to be independent predictors for need of organ dysfunction support in this population.

Risk scores AIMS65 and GBS were found to be both predictors for need of organ dysfunction support and AIMS65 had a higher accuracy in detecting these patients (AUC, 0,734; 95% CI 0,651-0,816) than GBS (AUC, 0,717; 95% CI 0,628-0,807). The four variables found in our study together showed more accuracy to predict need of organ dysfunction support (AUC, 0,911; 95% CI 0,873-0,949).

## Conclusions

Referring all patients with evidence of UGIB to the Emergency Room may be unnecessary and can prove to be costly and overwhelming to the capacity of the ER. AIMS65 and GBS may be a useful risk stratification tool to determine the level of care in ED, but the accuracy can be improved with new clinical and laboratory markers.

## References

[1] Blatchford O, Murray WR, Blatchford M (2000). A risk score to predict need for treatment for upper gastrointestinal haemorrhage. The Lancet.

